# The Relation of Dental Societies to the Profession

**Published:** 1891-01

**Authors:** Jos. King Knight

**Affiliations:** Boston, Mass.


### THE RELATION OF DENTAL SOCIETIES TO THE PROFESSION.[1]

BY JOS. KING KNIGHT, D.D.S., BOSTON, MASS.

When the chairman of our Executive Committee wrote me at literally the eleventh hour for a paper to be presented before this meeting, my first impulse was to decline for want of time and ability to prepare a suitable subject. But having been on this same committee, and remembering that the eleventh-hour invitations were sent out after all attempts had failed to secure talent of a better grade, and having all manner of sympathy for the committee, in a moment of weakness I yielded, and the Society must take the consequences.

Without attempting a finished paper, I have chosen to present to your attention a subject, with such few thoughts as may suggest themselves to me, in the hope that their introduction may start a line of discussion from which some good may result. I have not selected this theme because of any special fitness or preparation on my part, but because I deem it one which should engage our most serious attention at the present time.

The past decade has witnessed a most radical change in our profession, not only in the methods of operating, but in the plan of carrying on our business; for no one at this time will deny that while our profession is in many respects analogous to that of the medical fraternity, in many other aspects it is susceptible of being classed with the higher arts and sciences.

It is a profession which stands by itself; needs no bolstering up

---

[1] Read before the Massachusetts Dental Society, December, 1889.

from outside sources; cannot be regulated by the antique methods which govern some bodies which may have some features in common; needs and must have rules and regulations designed especially for it, and for the times in which we live, if it is proposed to keep the bright, active, intelligent, and inventive young men who are growing up in the ranks in harmony with the societies which claim to represent this profession.

My attention was called anew to this train of thought by an editorial in the INTERNATIONAL DENTAL JOURNAL for October, 1889, in which the writer says,—

"The past meeting of the Association at Saratoga was not up to former sessions held at that place. The attendance was much smaller and the interest in the proceedings was much less evidenced than at former meetings. Few papers were presented and the discussions were very limited, so much so that the editor of the *Cosmos* pronounced the meeting 'the least valuable of any held for several years.' . . . The facts in the case are that the profession is largely written out. As we said editorially in the August number of the JOURNAL when opposing the calling of an International Dental Congress, so say we again, 'Those who are directly connected with the American Dental Association realize the fact that it is growing harder every year to get suitable material to make a good programme; and why? Simply because of the division of interest on account of the many anniversary meetings of large proportions which have been held in America the past few years.' The American Dental Association is in its decadence, and unless something is done to revive interest in this time-honored institution it will surely pass into oblivion."

Referring to the same subject, a writer in the *Odontographic Journal* says,—

"There are two prominent disintegrating forces at work, and have been seriously undermining the foundations for a long period. They are, briefly, first, the stringency of constitutional enactments and the combined determination of a few learned in the law to enforce these to the bitter end, and, second, the annual effort to make this convention a political machine, in which the legitimate work of such a body is subordinated to the struggle of rival factions for the control of the presidency."

And what is true of the American Dental Association I fear may hold equally true of kindred organizations. Celebrating as we do to-day the twenty-fifth anniversary of this our beloved Society, it behooves us to consider the past, observe the present times, and, with a wise and fearless outlook into the future, so shape our course that the society shall take a new hold upon the members of the profession, and secure their hearty co-operation and support, if we expect it to grow and flourish until the silver shall become the golden year.

Let us look for a moment at what should be the relation sustained by the society to the profession, the faults in our present organization, and the remedies if we can discover them.

The society should be to the profession what the college is to the student; it should be a post-graduate course; a place for mutual conference and observation; a place where one may come to get and to give new ideas; a place for experimentation of those new or radical developments which may at the time be of general importance to the profession; a crucial test which may be applied to all new inventions, that those worthy of adoption may be accepted and the worthless or fraudulent thrown to one side. It should have the condensation and concentration of the experience of individual members, that valuable time may not be lost; and, lastly, it should be able to receive the presence of those eminent in skill and teaching to give by word of mouth that which cannot be so well communicated by the pen. And by these and all means made so interesting and instructive that every member of the profession would find it *more* profitable to be present than absent.

What now are some of the apparent failings in our present methods of conducting societies? I think we will all agree that there are too many different organizations, covering approximately the same field, and holding so many meetings that the interest and attendance is divided, with a consequent loss to all concerned. Take Massachusetts, for example. Out of over a thousand registered dentists, only about one in nine belong to this Society, about one in eighteen to the Connecticut Valley, and the same proportion in the New England, making in all three a little less than one in five who have any membership in these State organizations. And when we consider that many belong to two or all of these societies, we must admit that the proportion is much less, probably not greater than one in seven or eight.

It appears to me that the remedy for this state of affairs is to so systematize the work that there shall be district societies occupying a limited portion of the State, with meetings quarterly or oftener if desired; a State organization meeting once a year, taking one full day and not the parts of two days, *membership to which should be through the district societies;* and a national society, made up of representatives of the different State organizations, *with jurisdiction over all*, meeting for business and discussion of matters of great importance once in three years. Then the proceedings of these State and national societies should be of such general interest and magnitude as to demand publication in full; thus every member of the profession, from Maine to California, would need to belong to only one society to keep fully abreast with the times, and be in a position to come in personal contact with the best talent of our day. We do not realize the extent and resources of our country in this direction, nor the possibilities of good which would accrue from such a union of effort. We are aware that district societies exist in some sections of the country, but without acknowledging any allegiance to State or national authority. The spirit of the times is towards concentration, and I believe some such plan as this, thoroughly matured as to detail, would result in reducing the heterogeneous, go-as-you-please mass of individual, and ofttimes conflicting, societies into a homogeneous system, with a consequent benefit to the organizations and the profession.

It will be observed that one of the subjects announced for discussion is the formation of an Eastern Dental Association. It strikes me that it would be more to the purpose if the Massachusetts Dental Society would take the initiative, by the appointment of a committee of conference, which shall have for its object the endeavor to unite the many individual societies now in existence into a system or union, which would possess greatly increased power and influence.

Secondly, there is altogether too much waste of time at these gatherings as generally conducted. By attempting to spread them out over two or three days there is an uncertainty as to when any particular subject is to be discussed, and those who have a peculiar interest in that subject, rather than spend hours waiting for it, will absent themselves altogether. If one could attend the district meeting for an evening; could leave his office after the close of a day's work, spend the next day at the State gathering crowded full of valuable points, and be back at the office on the morning of the third; and for the national convention, once in three years, devote the entire week to an interchange of thought and outline work, I believe that all the meetings would gain in attendance and usefulness.

And this could be accomplished by doing away entirely with the reading of papers, having such as are approved by the Executive Committee printed and mailed to each member prior to the meeting, certain hours being assigned for their discussion at the convention, and reducing to a minimum the time used in the discharge of detail business and reports.

Not to weary you, I will say, in the third and last place, that I am impressed that our societies, as to-day organized, are too restrictive in their membership, taking the code of ethics as a test. In our endeavor to guard our ranks against the incompetent and impostor, and with a prejudice born of want of consideration on our part, we keep high the barriers which our forefathers raised, forgetting the fact that our age has outgrown the former standards, and that the competition which is so keen in business life has not left our sacred circle untouched.

Now, do not misunderstand me. There is a large and growing class of charlatans, whose sole object in entering the dental ranks seems to be the ready means of fleecing an unsuspecting public. And in our cities it is especially true that there is a large population who do not know where to turn for reliable dental service; nor are they, in many cases, competent to judge of the quality of the service which they receive. This may be said of many who would rank among the intelligent class. Now the empiric will blaze out in flaming advertisements and inducements of the most attractive kind, and all he wants of the dental societies (aside from stealing what information he can without helping to pay the bills) is that they will keep their code of ethics so strict that the members can in no way, by the use of printers' ink, enlighten the public, or trespass upon what he considers his particular stamping-ground. Thus far the charlatan and the old school dental society are agreed. A reference to Article XII. of our by-laws, for example, shows us that "it is unprofessional to resort to public advertisements, cards, handbills, posters, or signs *calling attention to peculiar styles of work*, lowness of prices, *special modes of operating; . . . to publish reports of cases* or certificates in the public prints." And other sections might be quoted if needed.

But are we dealing justly with all the younger members of the profession, who, while fully competent to do good work if they can obtain it, are privileged to sit hour after hour waiting for patients that rarely come, because, forsooth, they have been forbidden to let their light shine and announce to the world wanting such service their ability to perform the same? As Dr. Robinson says, "It is the right and duty of the dentist to inform the public in any legitimate way about his profession, provided he tells the truth and does not promise what he is unable to perform. It is an American privilege, and is in keeping with the push that is practised in every department of life." An article in the *Cosmos* for August has been brought to my attention since writing the above, and contains many valuable suggestions, but I cannot agree with the writer where he says, "There is a wide difference between a business and a profession. The one is narrow, grasping, self-centred, illiberal; the other broad, magnanimous, and liberal. Business cannot be conducted successfully on professional principles; neither can a profession be creditably conducted upon business principles. I do not wish to cast any reflection upon legitimate business methods. In this age of close competition only the shrewdest can survive; the liberal business-man will surely fail. It is, however, the duty of the dental society to see that its code of ethics rules out business principles as applied to the investigation of matters relating to the general welfare."

I cannot follow him there. I believe that, taken as a rule, the successful business-man will compare favorably in moral stamina and lofty aspirations with the best of the dental profession; and it is to me a cause of regret that we should attempt to assume the "holier-than-thou" attitude, and claim that disinterested philanthropy and unselfish desire to serve the "dear public" is a monopoly of the "liberal professions."

As I have said in the beginning of my remarks, our profession is different from every other and must stand upon its own merits. There is an inseparable union between the operative and the mechanical; between the art of healing and the trade of manufacturing and supplying artificial appliances; between the profession of deep research into the hidden causes of pathological conditions and their removal and the business of selling the best article we can produce to suit the purse and the needs of our patients. And when we as societies recognize this fact, and conform our regulations to the demands of the times and to the protection of all conscientious and consistent workmen, we will have taken a long stride in advance, and opened the door for the admission of just that class which is needed to give new life and energy to these fast-failing bodies. As one has said, "We cannot afford to lose so many of our progressive men as would be forced to leave if we put a strict construction upon the ethics as they are now in force among the medical and dental professions. Professions are made strong by what they *include* rather than by what they *exclude*."

Furthermore, with the idea of keeping our skirts free from any taint of barter or trade, we have declared in spirit, and sometimes in words, that it is unprofessional for one to avail himself of the protection which the government affords to those who, by the activity of their brains, may work out ideas worthy of protection. I know that this question of patents is a vexed and vexatious one, and no words of condemnation can be too strong for those who obtain the government's aid to enable them to impose upon the profession, either by the patenting of articles which are not original with them, or by the iniquitous system of license or royalty to place the profession under restrictions as to certain methods of procedure. But, on the other hand, where one has faithfully, and by the expenditure of time, brain, and money, produced an invention the general introduction of which will be a benefit to the profession at large, I claim that it is *unprofessional for the societies* to deny to their brother member the just recompense of his skill, and insist that he shall deliver the production of his brain over to the manufacturer, to be either an additional source of revenue to him or be stifled as inimical to some of his other interests.

These, gentlemen, are some of the thoughts, hurriedly jotted down, which have occurred to me, and I am compelled to ask the pardon of the society for presenting them in so crude a form. If anything which has been said shall tend to awaken discussion which shall result in broadening our borders and bringing within the beneficent influences of our societies a larger proportion of those who are working along the same line with us, yet who may not see the way clear to conscientiously uniting themselves in our iron-clad covenant, I shall be content.

